# Adherence to treat-to-target recommendations in juvenile idiopathic arthritis in clinical practice

**DOI:** 10.1186/s12969-026-01265-w

**Published:** 2026-09-23

**Authors:** S. De Roock, P. C. E. Hissink Muller, R. S. M. Yeung, S. J. Vastert, D. A. Marshall, S. M. Benseler, J. F. Swart

**Affiliations:** 1 Department of Pediatric Rheumatology/Immunology, Wilhelmina Children’s Hospital, University Medical Center Utrecht, Utrecht, The Netherlands; 2 Faculty of Medicine, Utrecht University, Utrecht, The Netherlands; 3 Pediatric Rheumatology, Leiden University Medical Center, Willem Alexander Children’s Hospital, Leiden, The Netherlands; 4 Division of Rheumatology, Department of Paediatrics, Immunology and Institute of Medical Science, The Hospital for Sick Children, University of Toronto, Toronto, ON Canada; 5 Center for Translational Immunology, University Medical Center Utrecht, Utrecht, The Netherlands; 6 Departments of Community Health Sciences and Medicine, Cumming School of Medicine and McCaig Institute for Bone and Joint Health, University of Calgary, Calgary, Canada; 7 Alberta Children’s Hospital Research Institute, University of Calgary, Calgary, Canada; 8 Division of Rheumatology, Department of Pediatrics, Alberta Children’s Hospital, Cumming School of Medicine, University of Calgary, Calgary, Canada

## Abstract

**Objective:**

To assess adherence to Treat-to-Target (T2T) recommendations for juvenile idiopathic arthritis (JIA) in clinical practice.

**Methods:**

A nested cohort study in the multicenter international prospective UCAN JIA cohort was performed. Patient characteristics, clinical features, classification, disease activity as measured by number of active joints (AJC), Physician Global Assessment (PhGA) and Patient Global Assessment (PPGA) and treatment at baseline and six-months visit was assessed. Data were compared to published, consensus-based T2T recommendations. Logistic regression with step-wise selected variables identified factors related to the decision to change therapy.

**Results:**

A total of 476 children were included, 62% female, median age was 10.0 years. At baseline, anti-inflammatory medications (NSAID) and/or intra-articular injections were started in 53%, conventional synthetic anti-rheumatic drugs (csDMARD) in 37% and biologic therapies in 11%. At six-months, 248 patients (52%) had inactive arthritis based on AJC. PPGA was not collected for 35% of patients. In children with arthritis, treatment change as suggested in the recommendations were only made in 33%. Patients that had no treatment change were more often already treated with a csDMARD or biological or had limited number of joints affected. Multivariate logistic regression showed that the decision to change treatment at six-months was significantly related to PhGA, prior treatment changes and use of csDMARD or biological since baseline.

**Conclusion:**

PPGA is not consistently collected, hampering measurement-based care and T2T. PhGA and prior treatment steps are underlying current treatment decisions, but often not according to the T2T cut-offs and time-points that have been agreed upon in consensus.

## Introduction

Juvenile Idiopathic Arthritis (JIA) is the most common chronic musculoskeletal disease in childhood characterized by inflammatory joint involvement including joint swelling and/or pain and limited range of motion [1]. Many children continue to have active arthritis and suffer from its sequalae as adults [2]. Patients are assigned to one of seven categories based on the 2001 International League against Rheumatism (ILAR) classification [3–5]. Importantly, selection and access to treatment is associated with the assigned ILAR category [6]. Control of joint inflammation is the primary goal of JIA drug therapy aiming to limit joint pain and swelling in the short term and prevent chronicity and joint damage long-term [7].

In the past, the JIA classification driven approach was lacking evidence based guidance and defined endpoints. In 2010, an international task force pioneered a novel strategy for adult rheumatoid arthritis defining an evidence based path of treatment selection and aiming to achieve and maintain the pre-defined treatment goals [8, 9]. Keys to this “treat-to-target” (T2T) strategy were (1) defined care goal setting, (2) iterative standardized care-integrated measurements of validated outcomes and (3) treatment adjustments guided by these results.

The task force not only iteratively updated its recommendation for adults; in 2018 the pediatric rheumatology community was engaged to establish T2T recommendations and guidance for optimal timing and measurement-based care in JIA [10]. These include a step-up approach from nonsteroidal anti-inflammatory medications (NSAIDs) to conventional synthetic disease modifying anti-rheumatic drugs (csDMARD) and biologic therapies for the majority of non-systemic JIA categories [1, 3, 11–13]. Optimal JIA patient outcomes include clinically inactive disease (CID), ideally sustained off medications [9]. The latter may be closely linked to rapidly achieving disease control and preventing disease progression [6]. Remission was defined as the main treatment target, with the alternative target of low disease activity. Furthermore, in all non-systemic JIA patients, at least a 50% improvement in disease activity should be reached within three months and the target within six-months. Iterative evaluations aim to ensure achievement and maintenance of the target and provide a robust measurement framework for health care teams and families [1, 10].

The recommendations have been adopted internationally and contextualized based on the performance of the health care systems and the availability of medications. The treat-to-target recommendations did not include an implementation or evaluation strategy; however the T2T impact has been documented in JIA research studies [14–16] and in one analysis of a clinical registry [17]. There remains a paucity of data from prospective, real-life cohorts focusing on adherence with JIA treatment recommendations and T2T approaches [18]. The real-life impact of adherence to these recommendations needs to be determined and barriers to achieving the proposed goals must be identified.

Therefore, the aims of this study were to (1) to determine the adherence to JIA T2T recommendations in an international, contemporary cohort of children at the six-months visit, and (2) to identify key factors representing barriers to T2T adherence and effective disease control.

## Methods

The international, multicenter prospective UCAN CAN-DU (Understanding Childhood Arthritis Network, Canada and The Netherlands) study enrolled consecutive children and youth with arthritis since April 2019. Children were included in this nested cohort study, if they (1) met criteria for JIA based on ILAR classification [5], (2) had evidence of active arthritis at initial presentation (baseline), (3) were treatment naive except for NSAIDs and (4) had a follow-up assessment after six-months (+/- 2 months). Children with the systemic JIA subtype were excluded from this analysis as treatment is considerably different [19]. Patients enrolled in the UCAN CAN-DU study were treated according to regular local clinical practice, there were no explicit instructions to follow T2T recommendations. Data were captured in the password protected, General Data Protection Regulation (GDPR) - compliant international UCAN eHealth platform. This study was performed in line with the principles of the Declaration of Helsinki. Reference ethical approvals were granted by the University of Calgary (Conjoint Health Research Ethics Board, REB17–1563) for Canada and by the Ethical Board of Utrecht (18–474) for the Netherlands.

### Clinical phenotype, inflammatory markers and medications

Demographic data included age at diagnosis and biological sex. Serial clinical phenotyping comprised the joint status including location and phenotype of arthritis. Medication data encompassed type, dose and frequency as well as start and stop dates. JIA diagnosis was physician reported according to ILAR classification [5]. Medication start or change was allocated to a visit when initiated within 31 days after the visit, since necessary insurance applications frequently delay actual start following the decision to start a new drug. Changes in dose or frequency were allocated to a visit when initiated within 7 days after the visit. For this analysis medication types were grouped into NSAID and/or IA-injections (including Celecoxib, Ibuprofen, Indomethacin and Naproxen), csDMARD (including Methotrexate, Leflunomide, Sulfasalazine) and biological therapy (including Adalimumab, Etanercept, Infliximab, Golimumab, Tocilizumab and Abatacept).

Medication changes were categorized as *increased dose* when frequency or dose was changed within a group of medication, as *step up* when medication was changed to a medication group with higher potency (e.g. from csDMARD to biological therapy), as *switch biological* when a different type of biological therapy was started, or as continued when none of the above was the case.

### Physician and patient reported outcome measures

Disease activity parameters included the active joint count (AJC), patient/parent derived global assessment (PPGA) of well-being and physician derived global estimate of disease activity (PhGA) both on a 10-point scale with 0.5-point increments. These elements, when all available, can be integrated into the clinical Juvenile Arthritis Disease Activity Score (cJADAS71) score [20]. Patients or parents were asked to report their PPGA on a mobile-app or web-based questionnaire at baseline and six-month visit.

### Definition of disease activity

Clinical inactive disease (CID) according to the treat-to-target recommendation was defined as cJADAS ≤ 1 [10]. Here, active arthritis was used for evaluating treatment decisions, as the composite score cJADAS was often not available due to missing PPGA. A cut-off of AJC = 0 was used for inactive arthritis and was compared to CID cJADAS ≤ 1 where PPGA was available. A possible relation between PPGA missingness and presence of arthritis (AJC > 0) was tested. The assumption that AJC > 0 would result in cJADAS > 1 was tested.

### Treat-to-target recommendations

Measurement-based care benchmarks were anchored in the proposed European League Against Rheumatism/ Pediatric Rheumatology European Society (EULAR/PRES) T2T Task Force Recommendations [10]. These define a treatment target of CID (cJADAS ≤ 1) for all JIA patients at six-months after start of treatment. Treatments are mandated to be adjusted until the target of CID is reached.

The recommendations define that in patients with active disease at six months defined as either cJADAS > 1 or AJC ≥ 1, treatment should have been or needs to be adjusted including dose and/or frequency change and/or step up of medication class.

All treatment modification between baseline and follow up were captured and linked to the six-month follow-up visit when effectuated between one week before or one month after the visit.

Observed treatment choices were compared to the T2T recommendations for six-months after treatment initiation, where treatment adjustment is warranted for patients with active arthritis. Patients with active arthritis that had continued or changed therapy were compared with regards to baseline and six-months characteristics.

### Analysis

Comparisons of non-parametric, numeric variables were made using Wilcoxon rank sum test. Dependency of variables on each other was tested using Pearson’s Chi-squared or Fisher’s exact test as indicated. A logistic regression model was estimated for patients with active arthritis at six-months, and for all patients. Treatment change (yes/no) at six-months was set as the dependent variable, and AJC, PhGA, baseline treatment, treatment change (yes/no) prior to the six-months visit and reduction in AJC between baseline and six-months as independent co-variates. A random intercept for country was added because access to biological DMARDs differs between Canada and the Netherlands. Backwards variable selection showed that removal of the variable time between treatment start and six-month visit improved the model (based on Akaike’s Information Criterion; AIC). Six-month AJC was removed from the model for patients with arthritis at six-months. The variable inflation factor was analyzed for the final model to check for multicollinearity of the co-variates. The six-month AJC was removed from the model for all patients because of collinearity with PhGA. All analyses were performed using R version 4.4.1 and tidyverse [21]. The regression model was created using the package lme4 [22].

## Results

### Baseline characteristics and therapy

A total of 476 children and youth with non-systemicJIA were included in the study (Table 1). The majority of children originated from the Netherlands (59%); overall, 62% were females. Median age was 10.0 years (interquartile range (IQR) 4.0–14.0). Most patients were diagnosed with an oligo-articular JIA, followed by Rheumatoid Factor negative (RF^-^) polyarthritis and enthesitis-related arthritis (ERA). Patients had a median number of 2 joints affected at baseline, accompanied with a median PhGA of 3.0. For 137 patients (29%) a baseline PPGA was not available and disease activity according to cJADAS could not be ascertained (Table 1). For the patients of whom PPGA was available, the median PPGA was 4.0 and median cJADAS 10.

**Table 1:** Baseline characteristics of included juvenile idiopathic arthritis patients

	*N* = 476
**Country (NL)**	283 (59%)
**Sex (Female)**	296 (62%)
**Age at baseline**	10.0 (4.0, 14.0)
**ILAR classification**	
ERA	57 (12%)
Oligo-articular	247 (52%)
Poly-articular RF-	110 (23%)
Poly-articular RF+	23 (4.8%)
Psoriatic Arthritis	23 (4.8%)
Undifferentiated	16 (3.4%)
**AJC (baseline)**	2 (1, 5)
**PhGA (baseline)**	3.0 (2.0, 4.0)
PPGA (baseline)*	4.0 (1.5, 6.1)
cJADAS71 (baseline)*	10 (6, 15)
* PPGA missing	137 (29%)
**Medication started at baseline**	
NSAID and/or IA-injections	250 (53%)
csDMARD	176 (37%)
Biological	50 (10%)

Legend: Number of patients with percentages of total or median value with interquartile range (Q1, Q3). Abbreviations: ILAR: International League of Associations for Rheumatology, ERA: Enthesitis Related Arthritis, RF: Rheumatoid Factor, AJC: Active Joint Count, PhGA: Physician Global Assessment, PPGA: Patient-parent Global Assessment, cJADAS: clinical Juvenile Idiopathic Arthritis Disease Activity Score

At baseline, 53% were prescribed NSAIDs or intra-articular injections, 37% conventional synthetic Disease-Modifying Anti-Rheumatic Drugs (csDMARDs) including methotrexate (MTX) or leflunomide and 10% biologic medications (Table 1). Of patients that start on biological medication, 34% had ERA and 40% poly-articular JIA (data not shown).

### Disease activity at six months of treatment related to baseline characteristics

At the six-month visit, 248/476 patients (52%) had inactive arthritis with an AJC of 0 (Table 2). Comparison of patients with active or inactive arthritis at six-months showed that a higher active joint count and a higher PhGA at baseline were significantly associated with active arthritis at six-months (*p* < 0.001). There was no difference in the baseline demographic characteristics of children who reached inactive arthritis compared to those who had ongoing active arthritis. Patients with oligoarticular disease and patients receiving NSAIDs and/or intra-articular injections in the first six-months were more likely to have inactive disease at six-months. In contrast, children with arthritis at six-months were more likely to have polyarticular disease and had been started on csDMARDs at baseline. Patients that reached a state of inactive arthritis at six-months had more time in days between the baseline visit and six-months visit, as well as time between the actual start of medication and disease assessment at six-months (Table 2).

**Table 2:** Characteristics of juvenile idiopathic arthritis patients stratified by active arthritis at six-months visit

	Arthritis *N* = 228 (48%)	No arthritis *N* = 248 (52%)	*p*-value
**Country (NL)**	127 (56%)	156 (63%)	0.11^#^
**Sex (Female)**	146 (64%)	150 (60%)	0.4^#^
**Age at baseline**	11.0 (5.0, 14.0)	10.0 (4.0, 14.0)	0.4^$^
**ILAR classification**			0.004^#^
ERA	27 (12%)	30 (12%)	
Oligo-articular	100 (44%)	147 (59%)	
Poly-articular RF-	63 (28%)	47 (19%)	
Poly-articular RF+	17 (7.5%)	6 (2.4%)	
Psoriatic Arthritis	14 (6.1%)	9 (3.6%)	
Undifferentiated	7 (3.1%)	9 (3.6%)	
**AJC (baseline)**	3 (2, 7)	2 (1, 4)	< 0.001^$^
**PhGA (baseline)**	3.0 (2.0, 5.0)	2.5 (1.5, 3.6)	< 0.001^$^
PPGA (baseline)*	4.3 (1.4, 6.3)	4.0 (1.6, 6.0)	0.5
cJADAS71 (baseline)*	11 (7, 17)	9 (5, 13)	0.004^$^
* PPGA missing	67 (29%)	70 (28%)	
**Medication started at baseline**			0.011^#^
NSAID and/or IA-injections	108 (47%)	142 (57%)	
csDMARD	100 (44%)	76 (31%)	
Biological	20 (8.8%)	30 (12%)	
**Medication use at six-months**			< 0.001^#^
NSAID and/or IA-injections	69 (30%)	111 (45%)	
csDMARD	115 (50%)	76 (31%)	
Biological	44 (19%)	61 (25%)	
**Days between baseline and six-month visit**	175 (154, 200)	186 (167, 208)	0.002^$^
**Days between start medication and six-month visit**	182 (157, 209)	192 (172, 215)	< 0.001^$^
six-months cJADAS > 1*	145/146 (99.3%)	70 /165 (42%)	< 0.001^#^
* PPGA missing	82 (36%)	83 (37%)	
**six-months AJC + PhGA > 1**	222 (97%)	18 (7.3%)	< 0.001^#^

Legend: Number of patients with percentages of total or median value with interquartile range (Q1, Q3). # Pearson’s Chi-squared test, $ Wilcoxon rank sum test. Abbreviations: ILAR: International League of Associations for Rheumatology, ERA: Enthesitis Related Arthritis, RF: Rheumatoid Factor, AJC: Active Joint Count, PhGA: Physician Global Assessment, PPGA: Patient-parent Global Assessment, cJADAS: clinical Juvenile Idiopathic Arthritis Disease Activity Score, NSAID: nonsteroidal anti-inflammatory drugs, IA: intra-articular, csDMARD: conventional synthetic disease modifying anti-rheumatic drugs

### Adherence to T2T recommendations

According to the T2T recommendations treatment should be based on shared decision making; disease activity should be assessed using (c)JADAS, including PPGA. At six-months visit PPGA was missing for 165 patients (35%, Supplemental Table 1). PPGA missingness was randomly distributed over patients with active or inactive arthritis at baseline (Wilcoxon rank sum *p* = 0.72) and six-months (Wilcoxon rank sum *p* = 0.69).

For only one patient (0.7%) with arthritis at six-months for whom PPGA and cJADAS was available had AJC > 0 without cJADAS > 1 (cJADAS = 1, PPGA = 0 and PGA = 0). For the other 99% of patients with arthritis (AJC > 0) and for whom a six-month cJADAS was available the cJADAS was > 1 (Table 2). For patients without active arthritis where full cJADAS was available 42% had a cJADAS > 1 and would have needed treatment adjustment according to the actual T2T cut-off but not in our alternative active arthritis only cut-off. Six patients with active arthritis had a PhGA of 0, whereas 18 patients without active arthritis had a PhGA > 1.

Patients with active arthritis therefore did not reach the recommended target; treatment adjustment was thus mandated following the “adjusted medication strategy” for 228 of 476 patients.

**Fig. 1:**
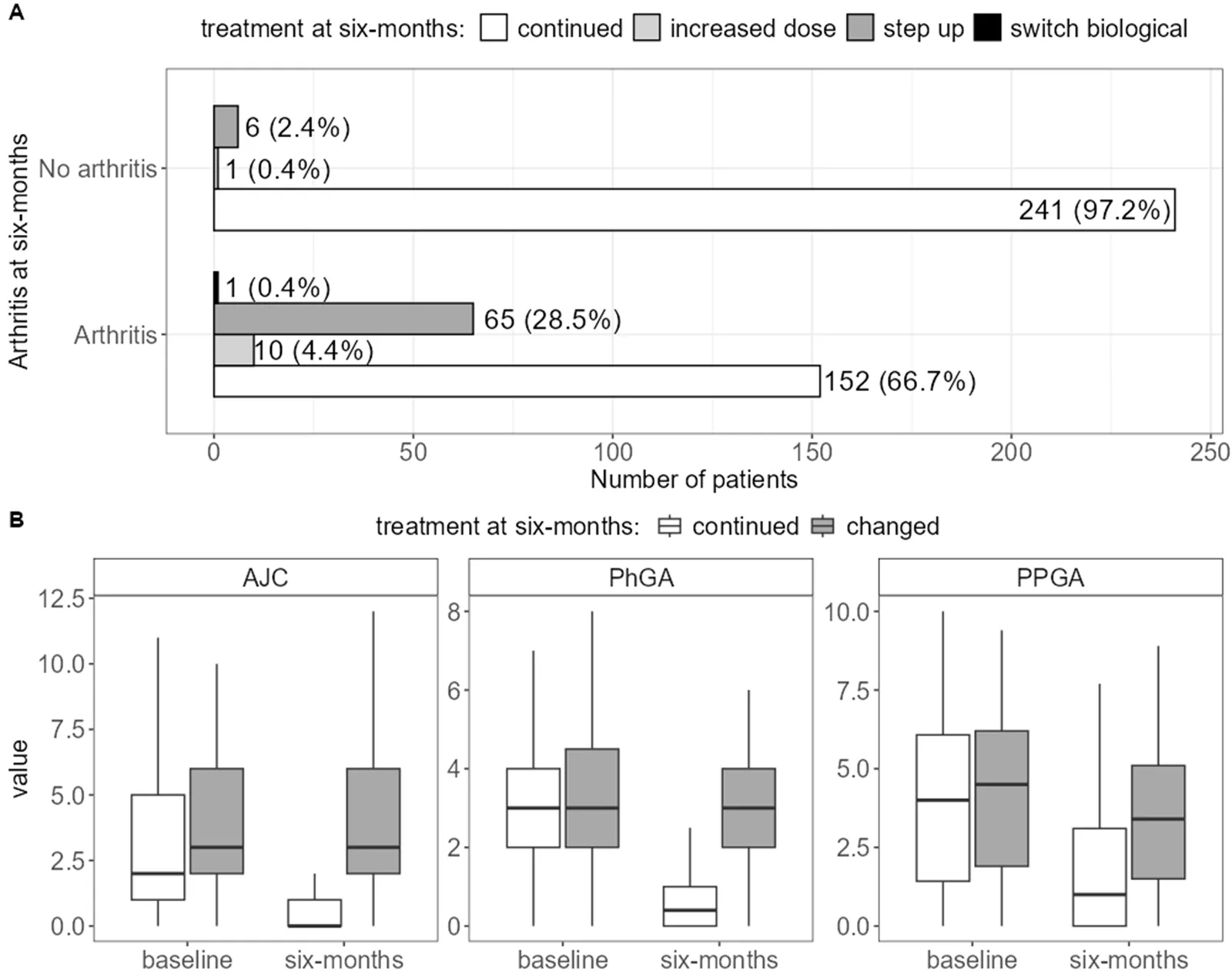
Treatment responses and decisions at six-months. **A**: Treatment decisions for patients with and without arthritis at six-months. **B**: Boxplots for Active Joint Count (AJC, *n* = 476: 393 continued therapy, 83 changed therapy), Physician Global Assessment (PhGA, *n* = 476: 393 continued therapy, 83 changed therapy) and Patient-parent Global Assessment (PhGA, *n* = 278/253 (continued therapy, baseline/six-months) and *n* = 61/58 (changed therapy, baseline/six-months)) at baseline and six-months visit for both patients that had continued treatment or changed treatment at six-months

However, 152 of the 228 children (67%) with arthritis at six-months were maintained on their treatment at the visit (Fig. 1A; Table 3). Some of these patients had a treatment change between baseline and six-months (51/152, 34%). A total of 101 patients had no change of treatment either before or at the six-month visit, while still having arthritis at six-months, representing 44% of the 228 patients.

**Table 3:** Baseline & six-months characteristics of juvenile idiopathic arthritis patients with active arthritis (*n* = 228) at six-month visit, stratified by continued or changed therapy at six-months

	Treatment at six-months		*p*-value
	continued therapy *N* = 152 (67%)	changed therapy *N* = 76 (33%)	
**Country (NL)**	75 (49%)	52 (68%)	0.006^#^
**Sex (Female)**	99 (65%)	47 (62%)	0.6^#^
**Age at baseline**	10.0 (4.0, 14.0)	11.0 (6.0, 14.0)	0.3^$^
**AJC (baseline)**	3.0 (1.0, 7.0)	3.5 (2.0, 7.0)	0.4^$^
**PhGA (baseline)**	3.00 (2.00, 5.00)	3.40 (2.00, 4.50)	0.6^$^
**PPGA (baseline)**	4.00 (1.40, 6.50)	4.50 (1.45, 6.00)	> 0.9^$^
PPGA missing	47 (31%)	20 (26%)	
**AJC (six months)**	1.0 (1.0, 3.0)	3.0 (2.0, 7.0)	< 0.001^$^
**PhGA (six months)**	1.50 (1.00, 2.50)	3.00 (2.00, 4.00)	< 0.001^$^
**PPGA (six months)**	1.85 (0.35, 4.00)	3.25 (1.50, 5.50)	0.057^$^
PPGA missing	60 (41%)	22 (28%)	
**Patients with mono-arthritis**	85 (56%)	11 (14%)	< 0.001^#^
**Reduction in AJC since baseline**	1.0 (0.0, 4.0)	0.0 (-2.0, 1.5)	< 0.001^$^
**Days between baseline and six-month visit**	177 (154, 203)	172 (152, 193)	0.086^$^
**Days between start medication and six-month visit**	185 (162, 211)	173 (148, 201)	0.017^$^
**Systemic medication started at baseline**			0.018^#^
NSAID and/or IA-injections	70 (46%)	38 (50%)	
csDMARD	63 (41%)	37 (49%)	
Biological	19 (13%)	1 (1.3%)	
**Treatment before six month visit**			0.051^#^
changed before six months	51 (34%)	16 (21%)	
continued since baseline	101 (66%)	60 (79%)	

Legend: Number of patients with percentages of total or median value with interquartile range (Q1, Q3). # Pearson’s Chi-squared test, $ Wilcoxon rank sum test. Abbreviations: ILAR: International League of Associations for Rheumatology, ERA: Enthesitis Related Arthritis, RF: Rheumatoid Factor, AJC: Active Joint Count, PhGA: Physician Global Assessment, PPGA: Patient-parent Global Assessment, cJADAS: clinical Juvenile Idiopathic Arthritis Disease Activity Score, NSAID: nonsteroidal anti-inflammatory drugs, IA: intra-articular, csDMARD: conventional synthetic disease modifying anti-rheumatic drugs

When adjustments were made at the six-month visit, “step-up” was the most frequently chosen approach in patients with arthritis (Fig. 1A). Medication remained unchanged for the vast majority of patients with no signs of arthritis at six-months (97%, Fig. 1A).

### Characteristics related to treatment change

The mean AJC, PhGA and PPGA of all patients reduced between baseline and six-month visit. Patients with a change in treatment at six-months had a more variable and less clear reduction in disease parameters between baseline and six-months (Fig. 1B, Supplemental Table 1). The AJC, PhGA and PPGA at six-months were all significantly higher for patients that had a change in treatment at six-months (*p* < 0.001, Wilcoxon rank sum test).

**Fig. 2:**
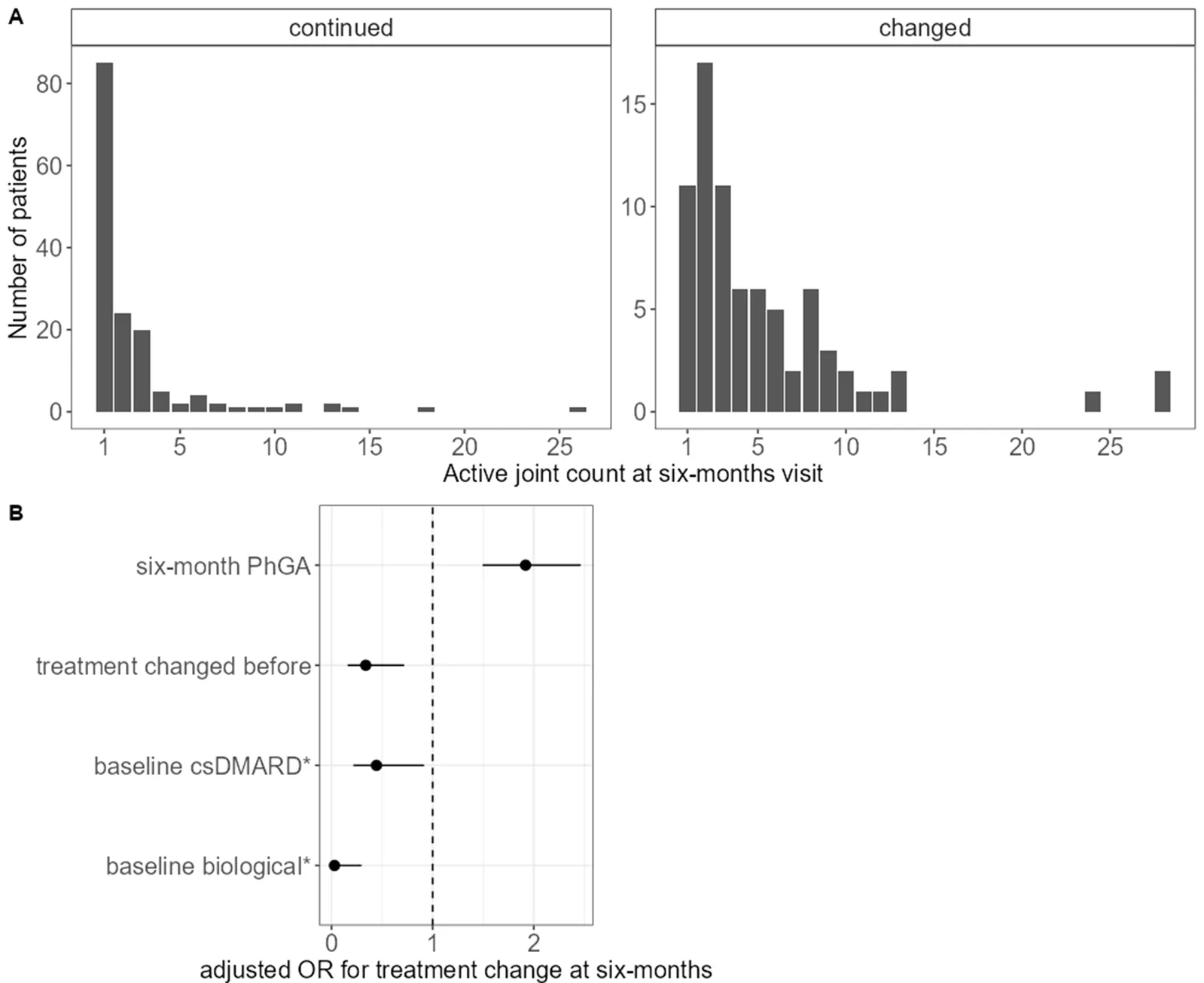
Number of joints affected by treatment decision and odds ratios for variables that influence the treatment decision at six-months. **A**: histograms for the number of affected joints at six-month visit for patients with active arthritis that had continued (left) or changed (right) treatment at six-month visit. **B**: Adjusted odds ratios (OR) for variables that influence the decision to change treatment at the six-months visit from multivariate logistic regression model for patients with active arthritis at six-months. The OR is adjusted for the other variables in the logistic regression model: six-month PhGA, treatment change before six-month visit, and baseline medication. *baseline csDMARD and baseline biological is relative to reference category baseline NSAID and/or IA-injections. NSAID: nonsteroidal anti-inflammatory drugs, IA: intra-articular, csDMARD: conventional synthetic disease modifying anti-rheumatic drugs, PhGA: Physician Global Assessment

For patients with active arthritis at six-months that did or did not have their treatment changed, AJC, PhGA and PPGA were comparable at baseline (Table 3). Patients with active arthritis at six-months that had no change in their treatment had a larger reduction in the AJC between baseline and six-months (Table 3). Moreover, these patients were more often left with arthritis of a single joint than patients that had a change in treatment (56% vs. 14%, Table 3; Fig. 2A). Patients with active arthritis that had no change in their treatment were also more often on biological treatment at baseline and/or had a prior change in treatment between study visits at baseline and six-month visit (Table 3).

PPGA and the composite cJADAS-score often could not have been used for treatment decisions by the physicians at six-months as PPGA was missing for 35% of patients. Multivariate logistic regression modeling to test which parameters influenced the decision to change medication in relation to each other included six-months AJC, six-months PhGA, medication started at baseline (NSAID and/or IA-injections, csDMARD or biologic), medication changes prior to the six-month visits and reduction in AJC between baseline and six-months. After backward step-wise selection based on AIC and correction for multicollinearity the model for patients with active arthritis only and for all patients contained PhGA, treatment change before six-months and baseline medication as independent variables.

The results showed that for patients with active arthritis at six-months, PhGA was significantly related to the decision to change treatment (adjusted odds ratio 1.9, p < < 0.001 (OR, adjusted for treatment change before six-months and baseline medication); Fig. 2B). Prior treatment changes (adjusted OR 0.34) and the use of csDMARD (adjusted OR 0.44) or biologic therapy (adjusted OR 0.03) before six-months compared to NSAIDs alone were related to not changing therapy at six-months (Fig. 2B). The model for all (patients with and without arthritis at six-months) showed comparable results (Supplemental Fig. 1).

## Discussion

In this prospective study to evaluate the adherence to JIA T2T recommendations across two health care systems a carefully selected set of patient-reported outcomes was provided to families. However, completion of the PPGA remained limited. Therefore, the T2T principle of shared decision making could often not be adhered to, and in that case, treatment decisions could not have been based on the (c)JADAS score.

T2T guidelines were not followed for the majority (65%) of patients with active arthritis at six-months. This is inline with a recently published analysis of a clinical registry where only 15% of patients with moderate to high disease activity had an escalation of therapy [17]. Although the reason for this cannot be concluded from the data, not changing the treatment was related to several characteristics. First, patients that already had a change in treatment before the six-months visit less often had a change at six-months again. The T2T guidelines do not explicitly mention the next steps after treatment has been changed at the three-months visit [10]. The data suggest that for most physicians the clock is reset to zero after treatment change and the treatment goal for the next three months is a 50% reduction in disease activity and not inactive arthritis. Second, patients that are already on a csDMARD or biologic since baseline less often have their medication changed: Physicians are more inclined to await the anticipated effect of the csDMARDs or biological treatment compared to NSAIDs and/or IA-injections and the six-month decision moment might come too soon. This is also reflected by the results of the multivariate logistic regression models presented here. Moreover, access to biological drugs can be limited by regulations, hampering the timely start of biologic therapy. Third, patients with active arthritis that had no change in treatment at six-months more often had limited arthritis or only one joint affected. The physician’s decision not to change treatment for these patients could reflect the idea that the disease status of these patients does not warrant csDMARD or biologic treatment. This is in line with a previously published study, where treatment escalation was mainly instigated in patients with increasing disease activity, and not for patients with stable active disease [17].

Our multivariable models show that the PhGA is the most relevant variable for physicians to change treatment. The active joint count alone was not part of the models as it did not add to the explained variation and showed collinearity with the PhGA. What other factors influence the PhGA score cannot be concluded from the data presented here. Extra-articular symptoms that influence treatment decisions are also relevant components of the PhGA score as shown before [23]. In another study it was concluded that disease worsening influences the decision to change treatment, not persistent disease activity [17]. Indeed, disease worsening can implicitly be reflected by the PhGA, but not by AJC.

Although treatment of the majority of patients in this study is not according to cut-offs and time-points agreed upon in T2T guidelines, a significant reduction of patients with active arthritis is observed at six-months. For most patients with active arthritis at six-months the number of affected joints is significantly reduced compared to baseline. Current treatment decisions therefore are effective for most patients. Nevertheless alignment with T2T time-points and cut-offs could improve the treatment results further. As recently shown, start of biological therapy early after symptom onset improves the response rate [24]. The time limit of six-months for reaching inactive disease could be experienced as relatively fast for most paediatric rheumatologist, specifically when improvement is observed within these six-months.

There were several limitations to the study. First, sample size was limited, and disproportional to the JIA populations of the two participating countries. This was reflecting the impact of distinctly different restrictions on clinical research imposed during the COVID-19 pandemic. The limited sample size relative to the number of variables in the regression model also warrants cautious interpretation of the presented odds ratios. Second, a long period between the decision to start therapy and the actual initiation of the therapy due to administrative issues for biologic therapy may result in shorter period on therapy than a six-month visit suggests. The results of this study indeed show that patients that had inactive arthritis at six-month visit had a longer median period on therapy. Nevertheless, the period on therapy was not related to the physician’s decision to change therapy for patients at six-month visit as it was removed from the logistic regression models during backward variable selection based on AIC.

A fourth limitation was the large number of missing PPGA. This made it impossible to analyze adherence to T2T recommendations based on the (c)JADAS composite score. As an alternative we analyzed disease only based on AJC with the a cut-off of 0 as inactive arthritis. The cut-off in the T2T recommendations for changing treatment at six-months is cJADAS ≤ 1 [8]. This cut-off will be exceeded with AJC = 1, as this will be accompanied by a PhGA > 0 and likely also a PPGA > 0. However, as a consequence the proportion of patients with inactive disease at six-months in our analysis could be higher than what we would have observed when cJADAS was used as disease score. PPGA has been shown to remain high in one out of four patients despite a low AJC [25]. For implementation of measurement-based care physicians should be aware to collect the PPGA as part of a questionnaire or at least as the single value.

In conclusion, current practice for JIA treatment changes is more influenced by the PhGA and prior treatment choices, than by the strict cut-offs in time and treatment response recommended by T2T protocols. PPGA is not consistently collected, hampering measurement-based care. Consideration will need to be given to the proposed T2T recommendations, the application of measurement-based care and the real-life barriers to adoption of these recommendations.

## Supplementary Information

Below is the link to the electronic supplementary material.

Supplementary Material 1

## Data Availability

The data that were used for this manuscript are available upon reasonable request.

## Appendix: Members of the UCAN CAN-DU and UCAN CURE Consortia

Members of the UCAN CAN-DU and UCAN CURE consortia include: Adam Huber, Bianca Lang, Chelsea DeCoste, Elizabeth Stringer, and Suzanne Ramsey (IWK Health Centre); Alan Rosenberg, Kate Neufeld, Mehul Jariwala, and Tristan Kerr (University of Saskatchewan); Alexander Mosoiu, Alisa Rachlis, Amy Xu, Arthur Cheng, Brenleigh Jebb, Brian Feldman, Bruno Pereira, Deborah Levy, Dilan Dissanayake, Elizaveta Limenis, Evelyn Rozenblyum, Harper Cheng, Jennifer Ji Young Lee, Lynn Spiegel, Rayfel Schneider, Ronald Laxer, Ruud Verstegen, Shirley Tse, and Trang Duong (The Hospital for Sick Children); Andrea Human, David Cabral, Herman Tam, Jaime Guzman, Kim Morishita, Kristin Houghton, Lori Tucker, Mercedes Chan, Ross Petty, and Tommy Gerschman (British Columbia Children's Hospital); Annet van Royen-Kerkhof, Berent Prakken, Erika Van Nieuwenhove, Marc Jansen, and Nico Wulffraat (Wilhelmina Children's Hospital, University Medical Center Utrecht); Ciarán Duffy, Nadia Luca, Roman Jurencak, and Tala El Tal (Children's Hospital of Eastern Ontario); Claire LeBlanc, Gaëlle Chédeville, Piya Lahiry, Rosie Scuccimarri, and Sarah Campillo (Montreal Children's Hospital); Clare Hutchinson (North York General Hospital); Daniah Basodan, Dax G. Rumsey, Hon Yan Ng, Jeanine McColl, Lillian Lim, and Tara McGrath (Stollery Children's Hospital); Danielle Brinkman; Elizabeth Legger and Wineke Armbrust (Beatrix Children's Hospital, University Medical Center Groningen); Ellen Schatorje and Esther Hoppenreijs (Amalia Children's Hospital, Radboudumc and Sint Maartenskinderkliniek); Elodie Boudes, Gillian Currie, Heinrike Schmeling, Muhammed Dhalla, Nicole Johnson, Paivi Miettunen, Ravneet Sran, and Rebeka Stevenson (University of Calgary); Erkan Demirkaya, Jonathan Park, and Roberta Berard (Children's Hospital, London Health Sciences Centre); Dieneke Schonenberg-Meinema, Giske Biesbroek, Mariken Gruppen and J. Merlijn van den Berg (Emma Children's Hospital, Amsterdam University Medical Centers); Gordon Soon (North York General Hospital/Health Sciences North); Joseph Cafazzo (Centre for Global eHealth Innovation, University Health Network, University of Toronto); Liane Heale, Michelle Batthish, and Tania Cellucci (McMaster Children's Hospital); Lily Lim (Children's Hospital Health Centre Winnipeg); Maarten IJzerman (Erasmus University and University of Melbourne); Marinka Twilt (University of Calgary and Alberta Children's Hospital); Marleen Verkaaik, Philomine van Pelt, and Sylvia Kamphuis (Sophia Children's Hospital, Erasmus University Medical Center); Michelle Kip (University of Twente); Nickolas Blanchette (Trillium Health Partners); Paul Dancey (Janeway Children's/Memorial University); and Regina de Geus (University Medical Center Utrecht).

## Declarations

### Patient and public involvement

The international multi-center UCAN CAN-DU study prospectively enrolls children with arthritis across Canada and the Netherlands. Patients and parents were involved in the writing of the UCAN CAN-DU grant and are involved in the research project.
