# Correction: Adherence to treat-to-target recommendations in juvenile idiopathic arthritis in clinical practice

**DOI:** 10.1186/s12969-026-01292-7

**Published:** 2026-11-15

**Authors:** S. De Roock, P. C. E. Hissink Muller, R. S. M. Yeung, S. J. Vastert, D. A. Marshall, S. M. Benseler, J. F. Swart

**Affiliations:** 1 Department of Pediatric Rheumatology/Immunology, Wilhelmina Children’s Hospital, University Medical Center Utrecht, Utrecht, The Netherlands; 2 Faculty of Medicine, Utrecht University, Utrecht, The Netherlands; 3 Pediatric Rheumatology, Leiden University Medical Center, Willem Alexander Children’s Hospital, Leiden, The Netherlands; 4 Division of Rheumatology, Department of Paediatrics, Immunology and Institute of Medical Science, The Hospital for Sick Children, University of Toronto, Toronto, ON Canada; 5 Center for Translational Immunology, University Medical Center Utrecht, Utrecht, The Netherlands; 6 Departments of Community Health Sciences and Medicine, Cumming School of Medicine and McCaig Institute for Bone and Joint Health, University of Calgary, Calgary, Canada; 7 Alberta Children’s Hospital Research Institute, University of Calgary, Calgary, Canada; 8 Division of Rheumatology, Department of Pediatrics, Alberta Children’s Hospital, Cumming School of Medicine, University of Calgary, Calgary, Canada

**Correction: Pediatric Rheumatology. 2026;24:67**

https://doi.org/10.1186/s12969-026-01265-w

Following publication of the original article [1], we have been notified that the collaborators names have not been correctly tagged under the authorship group.

The original article has been corrected.
