# Supplementary material for: Adherence to treat-to-target recommendations in juvenile idiopathic arthritis in clinical practice
Source: Pediatr Rheumatol Online J. 2026 Sep 23;24:67. doi: 10.1186/s12969-026-01265-w (PMC13632357; doi:10.1186/s12969-026-01265-w)
Supplement: Supplementary file 1 [file 12969_2026_1265_MOESM1_ESM.pdf]

Table 1: Baseline characteristics of included juvenile idiopathic arthritis patients

	**N = 476**
**Country (NL)**	283 (59%)
**Sex (Female)**	296 (62%)
**Age at baseline**	10.0 (4.0, 14.0)
**ILAR classification**	
ERA	57 (12%)
Oligo-articular	247 (52%)
Poly-articular RF-	110 (23%)
Poly-articular RF+	23 (4.8%)
Psoriatic Arthritis	23 (4.8%)
Undifferentiated	16 (3.4%)
**AJC (baseline)**	2 (1, 5)
**PhGA (baseline)**	3.0 (2.0, 4.0)
**PPGA (baseline)***	4.0 (1.5, 6.1)
**cJADAS71 (baseline)***	10 (6, 15)
* PPGA missing	137 (29%)
**Medication started at baseline**	
NSAID and/or IA-injections	250 (53%)
csDMARD	176 (37%)
Biological	50 (10%)

Legend: *Number of patients with percentages of total or median value with interquartile range (Q1, Q3). Abbreviations: ILAR: International League of Associations for Rheumatology, ERA: Enthesitis Related Arthritis, RF: Rheumatoid Factor, AJC: Active Joint Count, PhGA: Physician Global Assessment, PPGA: Patient-parent Global Assessment, cJADAS: clinical Juvenile Idiopathic Arthritis Disease Activity Score.*

Table 2: Characteristics of juvenile idiopathic arthritis patients stratified by active arthritis at six-months visit

	**Arthritis**<br>**N = 228 (48%)**	**No arthritis**<br>**N = 248 (52%)**	**p-value**
**Country (NL)**	127 (56%)	156 (63%)	0.11#
**Sex (Female)**	146 (64%)	150 (60%)	0.4#
**Age at baseline**	11.0 (5.0, 14.0)	10.0 (4.0, 14.0)	0.4$
**ILAR classification**			0.004#
ERA	27 (12%)	30 (12%)	
Oligo-articular	100 (44%)	147 (59%)	
Poly-articular RF-	63 (28%)	47 (19%)	
Poly-articular RF+	17 (7.5%)	6 (2.4%)	
Psoriatic Arthritis	14 (6.1%)	9 (3.6%)	
Undifferentiated	7 (3.1%)	9 (3.6%)	
**AJC (baseline)**	3 (2, 7)	2 (1, 4)	<0.001$
**PhGA (baseline)**	3.0 (2.0, 5.0)	2.5 (1.5, 3.6)	<0.001$
**PPGA (baseline)***	4.3 (1.4, 6.3)	4.0 (1.6, 6.0)	0.5
**cJADAS71 (baseline)***	11 (7, 17)	9 (5, 13)	0.004$
* PPGA missing	67 (29%)	70 (28%)	
**Medication started at baseline**			0.011#
NSAID and/or IA-injections	108 (47%)	142 (57%)	
csDMARD	100 (44%)	76 (31%)	
Biological	20 (8.8%)	30 (12%)	
**Medication use at six-months**			<0.001#
NSAID and/or IA-injections	69 (30%)	111 (45%)	
csDMARD	115 (50%)	76 (31%)	
Biological	44 (19%)	61 (25%)	
**Days between baseline and six-month visit**	175 (154, 200)	186 (167, 208)	0.002$
**Days between start medication and six-month visit**	182 (157, 209)	192 (172, 215)	<0.001$
**six-months cJADAS > 1***	145/146 (99.3%)	70 /165 (42%)	<0.001#
* PPGA missing	82 (36%)	83 (37%)	
**six-months AJC + PhGA > 1**	222 (97%)	18 (7.3%)	<0.001#

Legend: *Number of patients with percentages of total or median value with interquartile range (Q1, Q3). # Pearson's Chi-squared test, $ Wilcoxon rank sum test. Abbreviations: ILAR: International League of Associations for Rheumatology, ERA: Enthesitis Related Arthritis, RF: Rheumatoid Factor, AJC: Active Joint Count, PhGA: Physician Global Assessment, PPGA: Patient-parent Global Assessment, cJADAS: clinical Juvenile Idiopathic Arthritis Disease Activity Score, NSAID: nonsteroidal anti-inflammatory drugs, IA: intra-articular, csDMARD: conventional synthetic disease modifying anti-rheumatic drugs.*

Table 3: Baseline & six-months characteristics of juvenile idiopathic arthritis patients with active arthritis (n=228) at six-month visit, stratified by continued or changed therapy at six-months

	Treatment at six-months		
	**continued therapy N = 152 (67%)**	**changed therapy N = 76 (33%)**	**p-value**
**Country (NL)**	75 (49%)	52 (68%)	0.006#
**Sex (Female)**	99 (65%)	47 (62%)	0.6#
**Age at baseline**	10.0 (4.0, 14.0)	11.0 (6.0, 14.0)	0.3$
**AJC (baseline)**	3.0 (1.0, 7.0)	3.5 (2.0, 7.0)	0.4$
**PhGA (baseline)**	3.00 (2.00, 5.00)	3.40 (2.00, 4.50)	0.6$
**PPGA (baseline)**	4.00 (1.40, 6.50)	4.50 (1.45, 6.00)	>0.9$
PPGA missing	47 (31%)	20 (26%)	
**AJC (six months)**	1.0 (1.0, 3.0)	3.0 (2.0, 7.0)	<0.001$
**PhGA (six months)**	1.50 (1.00, 2.50)	3.00 (2.00, 4.00)	<0.001$
**PPGA (six months)**	1.85 (0.35, 4.00)	3.25 (1.50, 5.50)	0.057$
PPGA missing	60 (41%)	22 (28%)	
**Patients with mono-arthritis**	85 (56%)	11 (14%)	<0.001#
**Reduction in AJC since baseline**	1.0 (0.0, 4.0)	0.0 (-2.0, 1.5)	<0.001$
**Days between baseline and six-month visit**	177 (154, 203)	172 (152, 193)	0.086$
**Days between start medication and six-month visit**	185 (162, 211)	173 (148, 201)	0.017$
**Systemic medication started at baseline**			0.018#
NSAID and/or IA-injections	70 (46%)	38 (50%)	
csDMARD	63 (41%)	37 (49%)	
Biological	19 (13%)	1 (1.3%)	
**Treatment before six month visit**			0.051#
changed before six months	51 (34%)	16 (21%)	
continued since baseline	101 (66%)	60 (79%)	

Legend: *Number of patients with percentages of total or median value with interquartile range (Q1, Q3). # Pearson's Chi-squared test, $ Wilcoxon rank sum test. Abbreviations: ILAR: International League of Associations for Rheumatology, ERA: Enthesitis Related Arthritis, RF: Rheumatoid Factor, AJC: Active Joint Count, PhGA: Physician Global Assessment, PPGA: Patient-parent Global Assessment, cJADAS: clinical Juvenile Idiopathic Arthritis Disease Activity Score, NSAID: nonsteroidal anti-inflammatory drugs, IA: intra-articular, csDMARD: conventional synthetic disease modifying anti-rheumatic drugs.*

Supplemental Table 1: Baseline & six-months characteristics of ALL juvenile idiopathic arthritis patients at six-month visit, stratified by continued or changed therapy at six-months

	**Treatment at six-months**		
	**continued therapy N = 393 (83%)**	**changed therapy N = 83 (17%)**	**p-value**
**Country (NL)**	228 (58%)	55 (66%)	0.2#
**Sex (Female)**	246 (63%)	50 (60%)	0.7#
**Age at baseline**	10.0 (4.0, 14.0)	11.0 (6.0, 14.0)	0.2$
**AJC (baseline)**	2.0 (1.0, 5.0)	3.0 (2.0, 6.0)	0.035$
**PhGA (baseline)**	3.00 (2.00, 4.00)	3.00 (2.00, 4.50)	0.027$
**PPGA (baseline)**	4.00 (1.40, 6.10)	4.50 (1.90, 6.20)	0.46$
PPGA missing	115 (29%)	22 (27%)	
**AJC (six months)**	0.00 (0.00, 1.00)	3.00 (2.00, 6.00)	<0.001$
**PhGA (six months)**	0.40 (0.00, 1.00)	3.00 (2.00, 4.00)	<0.001$
**PPGA (six months)**	1.00 (0.00, 3.10)	3.40 (1.50, 5.10)	<0.001$
PPGA missing	140 (36%)	25 (30%)	
**Reduction in AJC since baseline**	2.0 (1.0, 4.0)	0.0 (-1.0, 2.0)	<0.001$
**Days between baseline and six-month visit**	183 (162, 203)	175 (150, 196)	0.007$
**Days between start medication and six-month visit**	189 (168, 213)	175 (147, 203)	<0.001$
**Systemic medication started at baseline**			0.004#
NSAID and/or IA-injections	207 (53%)	43 (52%)	
csDMARD	137 (35%)	39 (47%)	
Biological	49 (12%)	1 (1.2%)	
**Treatment before six month visit**			0.14#
changed before six months	106 (27%)	16 (19%)	
continued since baseline	287 (73%)	67 (81%)	

Legend: Number of patients with percentages of total or median value with interquartile range (Q1, Q3). # Pearson's Chi-squared test, $ Wilcoxon rank sum test. Abbreviations: ILAR: International League of Associations for Rheumatology, ERA: Enthesitis Related Arthritis, RF: Rheumatoid Factor, AJC: Active Joint Count, PhGA: Physician Global Assessment, PPGA: Patient-parent Global Assessment, cJADAS: clinical Juvenile Idiopathic Arthritis Disease Activity Scale, NSAID: nonsteroidal anti-inflammatory drugs, IA: intra-articular, csDMARD: conventional synthetic disease modifying anti-rheumatic drugs.
